# On the Mechanisms of the Protective Effect of Infections on Type 1 Diabetes

**DOI:** 10.1080/17402520400004557

**Published:** 2004

**Authors:** Hélène Feillet, Jean-François Bach

## Abstract

The incidence of type 1 diabetes (T1D) shows a worrying tendency for a steady increase in Western countries. Along the line of the hygiene hypothesis, evidence accumulates to suggest that this increase is explained by the decrease of infections due to improved hygiene and medical care. This article presents a review of epidemiological data and of the main putative underlying cellular and molecular mechanisms.


*Clinical & Developmental Immunology*, September/December 2004, Vol. 11 (3/4), pp. 191–194

Taylor & Francis healthsciences

### On the Mechanisms of the Protective Effect of Infections on Type 1 Diabetes

HÉLÈNE FEILLET and JEAN-FRANÇOIS BACH*

*Necker Hospital, INSERM U580,161 rue de Sèvres, 75015 Paris, France*

The incidence of type 1 diabetes (T1D) shows a worrying tendency for a steady increase in Western countries. Along the line of the hygiene hypothesis, evidence accumulates to suggest that this increase is explained by the decrease of infections due to improved hygiene and medical care. This article presents a review of epidemiological data and of the main putative underlying cellular and molecular mechanisms.

### EPIDEMIOLOGY

The steady rise in the incidence of T1D in developed countries over the last three decades is a matter of major public health concern (Gale, 2002). Taken together with a very uneven distribution of the disease worldwide, with a North–South gradient, this increase in incidence prompted epidemiologists to determine the factors that could possibly explain this unfortunate trend. The fact that similar epidemiological features have been observed for other autoimmune diseases, notably multiple sclerosis, and for allergic diseases (Bach, 2002), suggests that the explanation is probably not essentially specific to T1D. There is epidemiological evidence for the causal role of the decrease of infections in the increase of incidence of T1D.

The negative relationship observed between the decline in the incidence of major infectious diseases and increase in T1D is suggestive but not by any means demonstrative of a causal relationship. Similarly, the uneven geographical distribution of the disease shows interesting but only very indirect evidence in this direction, even though the increased T1D incidence in children in families having migrated from low-incidence to high-incidence countries (Bodansky *et al.*, 1992) is highly suggestive. Clearly northern countries have a higher socio-economic level than southern countries as is illustrated by the positive correlation observed between T1D incidence and gross national product in Europe (Bach, 2002). However, other factors differ among these various countries, such as climate and diet.

### CAUSAL RELATIONSHIP

Two sets of data, however, incriminate infections or more generally the infectious environment (sanitary quality of water and food, conditions of lodging). First, in studies in well-circumscribed regions such as Northern Ireland, a positive correlation was observed between socio-economic indices and T1D incidence with a particular implication of household crowding (Patterson *et al.*, 1996). Secondly, it is striking to note that T1D is more common in first-born children of families at risk for T1D, suggesting that for a given genetic inheritance, the exposure of children to infections brought by siblings may be protective (Deschamps *et al.*, 1986).

Other pieces of evidence for a causal relationship are derived from immune disorders other than T1D. The increased risk of atopic diseases in children systematically treated with antihelminthic agents (in an infected region) (Lynch *et al.*, 1993) or after vaccination against *Streptococcus pneumoniae* (Klugman *et al.*, 2003) is highly suggestive as well as protection from atopy afforded by administration or probiotics (Kalliomaki *et al.*, 2001).

In fact, the best evidence for a causal relationship is derived from animal models. Both the non-obese diabetic (NOD) mouse and the Bio-Breeding (BB) rat show T1D with a much higher incidence when they are bred in specific pathogen-free (SPF) than in conventional conditions (Bach, 2002). It is sufficient to decontaminate the mice bred in the latter type of environment to observe increased incidence, as has been well-demonstrated for NOD mice (Bach, 2002).

Even more striking is the prevention of T1D observed in NOD mice and to a lesser extent in BB rats by infection with various pathogens. The first demonstration of this protective effect in the context of T1D was the observation that administration of complete Freund's adjuvant to young NOD mice prevents diabetes onset (Sadelain *et al.*, 1990). NOD mice do not develop T1D when they are infected at an early age by bacteria such as

*Corresponding author. Tel.: +33-1-4449-5373. Fax: +33-1-4306-2388. E-mail: bach@necker.fr

ISSN 1740-2522 print/ISSN 1740-2530 online © 2004 Taylor & Francis Ltd
DOI: 10.1080/17402520400004557

*Mycobacterium bovis* (BCG) (Harada *et al.*, 1990) or *Mycobacterium avium* (Bras and Aguas, 1996), viruses such as lymphochoriomeningitis virus (Oldstone, 1990), lactodehydrogenase virus (Takei *et al.*, 1992) or murine hepatitis virus (Wilberz *et al.*, 1991), or parasites (such as schistosomes) (Cooke *et al.*, 1999).

It is interesting to note that the pathogen does not necessarily need to be alive to afford protection, as illustrated by the protective effect of bacterial extracts (streptococci, Toyota *et al.*, 1986; klebsiella, Sai and Rivereau, 1996 or *Escherichia coli*, our unpublished data).

### MECHANISMS

Animal models offer a remarkable way to dissect the mechanisms explaining the protective effect of infections on T1D.

### Homeostatic Competition

It had been known for several decades that successive administration of two unrelated antigens led to major interference with decreased response to the second antigen (Liacopoulos and Ben-Efraim, 1975). The mechanisms of antigenic competition are still ill-defined. They may involve competition for macrophage antigen uptaking or processing, notably for the binding of peptides to MHC molecules as has been described in some models.

Another approach to antigen competition is derived from the improved understanding of lymphocyte homeostasis. It had been known for a long time that lymphocyte depletion, as may be induced for example by administering large doses of glucocorticoids, is rapidly corrected independently by seeding of newly formed lymphocytes from the thymus. It has recently been realized that this lymphocyte expansion was driven by various signals including TCR-mediated recognition of self-peptides in the context of MHC molecules and various cytokines including IL-7 (Jameson, 2002).

The control of T cells involved in the pathogenesis of immune disorders by homeostatic mechanisms was suspected because of the common occurrence of autoimmune diseases in lymphopenic animals; whether lymphopenia is genetically determined in the case of BB rats (Poussier *et al.*, 1982), or experimentally induced as in the case of autoimmune states induced by thymectomy (Asano *et al.*, 1996) and/or sublethal irradiation (Fowell and Mason, 1993). It was thus particularly interesting to see that prevention of colitis developing in lymphopenic SCID mice reconstituted by CD4 T cells depleted from the regulatory subset of CD45RB$^{low}$ T cells is inhibited by expansion in the recipient mice of a T cell clone specific to an unrelated antigen (Barthlott *et al.*, 2003). Such inhibition of colitis development is most likely explained by competition for homeostatic signals between effector cells inducing colitis and unrelated rapidly proliferating lymphocytes. One may hypothesize that the lymphocyte expansion associated with an anti-infectious response could be at the origin of a similar competition. This is illustrated by the recent observation that administration of complete Freund's adjuvant in NOD mice is associated with a decreased proliferation of lymphocytes in response to homeostatic signals (King *et al.*, 2004).

### Bystander Suppression

Immune responses to infectious agents give rise to the generation of regulatory T cells like other immune responses. The suppression exerted by these regulatory T cells (which is specifically induced by the antigens of the pathogen) is likely to extend to other immune responses, according to the well-established phenomenon of bystander suppression mediated by regulatory cytokines such as IL-10 and TGFβ (Fowell and Mason, 1993). Evidence in this direction derives from studies indicating a role for these cytokines in the protective effect of infections in T1D. It has thus been reported that the diabetes protection afforded by CFA in NOD mice is abrogated by combined administration of anti-IL-4 and anti-IL-10 antibodies (Shehadeh *et al.*, 1993). This observation must, however, be interpreted with caution since CFA still protects NOD IL-4$^{-/-}$ and NOD IL-10$^{-/-}$ NOD mice from diabetes (whereas it does not in IFN$\gamma^{-/-}$ NOD mice) (Serreze *et al.*, 2001). In our laboratory, we observed that the protective effect of an *E. Coli* extract was abrogated by administration of a neutralizing anti-TGFβ antibody (in preparation). As far as NKT cells are concerned, one may mention here recent studies showing increased IL-10 production and NKT cell production in NOD mice protected from T1D by infestation with schistosomes (Zaccone *et al.*, 2003).

### NON-ANTIGEN RELATED SUPPRESSION. THE ROLE OF TOLL-LIKE RECEPTOR (TLR) STIMULATION

Infectious agents may also downregulate immune responses through mechanisms which do not involve the induction of an immune response against their antigenic constituents. The first mechanism to be considered in this context is that of TLR stimulation. One might assume that TLR stimulation essentially leads to the production of pro-inflammatory cytokines that could be expected to accelerate T1D progression. In fact, this is not what was observed with various TLR agonists. T1D onset is prevented in NOD mice by LPS and TLR4 agonists (Tian *et al.*, 2001), and by CpG, a TLR9 agonist (Quintana *et al.*, 2000). These observations are similar to those made in experimental colitis, where the protective effect of probiotics is not observed in TLR 9$^{-/-}$ mice (Rachmilewitz *et al.*, 2004).

Other mechanisms could be proposed as suggested by recent data obtained for the hepatitis A virus (which stimulates Th2 cells expressing the HAV receptor)

(McIntire *et al.*, 2001) and immunosuppression by a measles virus protein (Marie *et al.*, 2001) and by various parasites (Mazingue *et al.*, 1986).

### WHY DO INFECTIONS STIMULATE OR PROTECT FROM AUTOIMMUNITY?

There is well-documented evidence showing that infections may induce autoimmune diseases either through cross-reactivity with autoantigens (as in the case of rheumatic fever or Guillain-Barré syndrome) or by local inflammation (as in the case of Theiler's disease). There are still limited data in T1D for these two hypotheses even if epidemiological data have been reported in favor of certain viruses, particularly enteroviruses (notably Coxsackie B4) (Jun and Yoon, 2003). Perhaps the long lag time spent between the triggering infection and T1D clinical onset renders the finding of a clearer picture difficult.

In any case, the mechanisms leading to autoimmune disease induction (antigen mimicry or inflammation) and to protection (homeostatic competition, bystander suppression, TLR stimulation) are very different and not mutually exclusive. One may hypothesize that autoimmune diseases such as T1D may be triggered (or exacerbated) by specific pathogens, whether the specificity is due to epitopes cross reactive with β cell-specific autoantigens or to the organ tropism of the infectious agent (as in the case of Cocksackie B4 for the pancreas). Conversely, one may assume that the protective effect of infections is essentially non-specific in terms of the target organ of the autoimmune disease.

### *References*

Asano, M., Toda, M., Sakaguchi, N. and Sakaguchi, S. (1996) "Autoimmune disease as a consequence of developmental abnormality of a T cell subpopulation", *J. Exp. Med.* **184**, 387–396.

Bach, J.F. (2002) "The effect of infections on susceptibility to autoimmune and allergic diseases", *N. Engl. J. Med.* **347**, 911–920.

Barthlott, T., Kassiotis, G. and Stockinger, B. (2003) "T cell regulation as a side effect of homeostasis and competition", *J. Exp. Med.* **197**, 451–460.

Bodansky, H.J., Staines, A., Stephenson, C., Haigh, D. and Cartwright, R. (1992) "Evidence for an environmental effect in the aetiology of insulin dependent diabetes in a transmigratory population", *Br. Med. J.* **304**, 1020–1022.

Bras, A. and Aguas, A.P. (1996) "Diabetes-prone NOD mice are resistant to Mycobacterium avium and the infection prevents autoimmune disease", *Immunology* **89**, 20–25.

Cooke, A., Tonks, P., Jones, F.M., O'Shea, H., Hutchings, P., Fulford, A.J. and Dunne, D.W. (1999) "Infection with *Schistosoma mansoni* prevents insulin dependent diabetes mellitus in non-obese diabetic mice", *Parasite Immunol.* **21**, 169–176.

Deschamps, I., Lestradet, H., Busson, M. and Hors, J. (1986) "Effects of HLA genotype, age and birth order on empirical risk estimates for insulin-dependent diabetes in siblings of diabetic children. An actuarial evaluation", *Diabetes Res.* **3**, 391–396.

Fowell, D. and Mason, D. (1993) "Evidence that the T cell repertoire of normal rats contains cells with the potential to cause diabetes. Characterization of the CD4+ T cell subset that inhibits this autoimmune potential", *J. Exp. Med.* **177**, 627–636.

Gale, E.A.M. (2002) "The rise of childhood type 1 diabetes in the 20th century", *Diabetes* **51**, 3353–3361.

Harada, M., Kishimoto, Y. and Makino, S. (1990) "Prevention of overt diabetes and insulitis in NOD mice by a single BCG vaccination", *Diabetes Res. Clin. Pract.* **8**, 85–89.

Jameson, S.C. (2002) "Maintaining the norm: T-Cell homeostasis", *Nat. Rev. Immunol.* **2**, 547–556.

Jun, H.S. and Yoon, J.W. (2003) "A new look at viruses in type 1 diabetes", *Diabetes Metab. Res. Rev.* **19**, 8–31.

Kalliomaki, M., Salminen, S., Arvilommi, H., Kero, P., Koskinen, P. and Isolauri, E. (2001) "Probiotics in primary prevention of atopic disease: a randomised placebo-controlled trial", *Lancet* **357**, 1076–1079.

King, C., Ilic, A., Koelsch, K. and Sarvetnick, N. (2004) "Homeostatic expansion of T cells during immune insufficiency generates autoimmunity", *Cell* **117**, 265–277.

Klugman, K.P., Madhi, S.A., Huebner, R.E., Kohberger, R., Mbelle, N. and Pierce, N. (2003) "A trial of a 9-valent pneumococcal conjugate vaccine in children with and those without HIV infection", *N. Engl. J. Med.* **349**, 1341–1348.

Liacopoulos, P. and Ben-Efraim, S. (1975) "Antigenic competition", *Prog. Allergy* **18**, 97–204.

Lynch, N.R., Hagel, I., Perez, M., Di Prisco, M.C., Lopez, R. and Alvarez, N. (1993) "Effect of anthelmintic treatment on the allergic reactivity of children in a tropical slum", *J. Allergy Clin. Immunol.* **92**, 404–411.

Marie, J.C., Kehren, J., Trescol-Biemont, M.C., Evlashev, A., Valentin, H., Walzer, T., Tedone, R., Loveland, B., Nicolas, J.F., Rabourdin-Combe, C. and Horvat, B. (2001) "Mechanism of measles virus-induced suppression of inflammatory immune responses", *Immunity* **14**, 69–79.

Mazingue, C., Stadler, B.M., Quatannens, B., Capron, A. and de Weck, A. (1986) "Schistosome-derived inhibitory factor: an immunosuppressive agent preferentially active on T lymphocytes", *Int. Arch. Allergy Appl. Immunol.* **80**, 347–354.

McIntire, J.J., Umetsu, S.E., Akbari, O., Potter, M., Kuchroo, V.K., Barsh, G.S., Freeman, G.J., Umetsu, D.T. and Dekruyff, R.H. (2001) "Identification of Tapr (an airway hyperreactivity regulatory locus) and the linked Tim gene family", *Nat. Immunol.* **2**, 1109–1116.

Oldstone, M.B. (1990) "Viruses as therapeutic agents. I. Treatment of nonobese insulin-dependent diabetes mice with virus prevents insulin-dependent diabetes mellitus while maintaining general immune competence", *J. Exp. Med.* **171**, 2077–2089.

Patterson, C.C., Carson, D.J. and Hadden, D.R. (1996) "Epidemiology of childhood IDDM in Northern Ireland 1989–1994: low incidence in areas with highest population density and most household crowding. Northern Ireland Diabetes Study Group", *Diabetologia* **39**, 1063–1069.

Poussier, P., Nakhooda, A.F., Falk, J.A., Lee, C. and Marliss, E.B. (1982) "Lymphopenia and abnormal lymphocyte subsets in the "BB" rat: relationship to the diabetic syndrome", *Endocrinology* **110**, 1825–1827.

Quintana, F.J., Rotem, A., Carmi, P. and Cohen, I.R. (2000) "Vaccination with empty plasmid DNA or CpG oligonucleotide inhibits diabetes in nonobese diabetic mice: Modulation of spontaneous 60-kDa heat shock protein autoimmunity", *J. Immunol.* **165**, 6148–6155.

Rachmilewitz, D., Katakura, K., Karmeli, F., Hayashi, T., Reinus, C., Rudensky, B., Akira, S., Takeda, K., Lee, J., Takabayashi, K. and Raz, E. (2004) "Toll-like receptor 9 signaling mediates the anti-inflammatory effects of probiotics in murine experimental colitis", *Gastroenterology* **126**, 520–528.

Sadelain, M.W., Qin, H.Y., Lauzon, J. and Singh, B. (1990) "Prevention of type I diabetes in NOD mice by adjuvant immunotherapy", *Diabetes* **39**, 583–589.

Sai, P. and Rivereau, A.S. (1996) "Prevention of diabetes in the nonobese diabetic mouse by oral immunological treatments. Comparative efficiency of human insulin and two bacterial antigens, lipopolysacharide from *Escherichia coli* and glycoprotein extract from *Klebsiella pneumoniae*", *Diabetes Metab.* **22**, 341–348.

Serreze, D.V., Chapman, H.D., Post, C.M., Johnson, E.A., Suarez-Pinzon, W.L. and Rabinovitch, A. (2001) "Th1 to Th2 cytokine shifts in nonobese diabetic mice: Sometimes an outcome, rather than the cause, of diabetes resistance elicited by immunostimulation", *J. Immunol.* **166**, 1352–1359.

Shehadeh, N.N., La Rosa, F. and Lafferty, K.J. (1993) "Altered cytokine activity in adjuvant inhibition of autoimmune diabetes", *J. Autoimmun.* **6**, 291–300.

Takei, I., Asaba, Y., Kasatani, T., Maruyama, T., Watanabe, K., Yanagawa, T., Saruta, T. and Ishii, T. (1992) "Suppression of development of diabetes in NOD mice by lactate dehydrogenase virus infection", *J. Autoimmun.* **5**, 665–673.

Tian, J., Zekzer, D., Hanssen, L., Lu, Y., Olcott, A. and Kaufman, D.L. (2001) "Lipopolysaccharide-activated B cells down-regulate Th1 immunity and prevent autoimmune diabetes in nonobese diabetic mice", *J. Immunol.* **167**, 1081–1089.

Toyota, T., Satoh, J., Oya, K., Shintani, S. and Okano, T. (1986) "Streptococcal preparation (OK-432) inhibits development of type I diabetes in NOD mice", *Diabetes* **35**, 496–499.

Wilberz, S., Partke, H.J., Dagnaes-Hansen, F. and Herberg, L. (1991) "Persistent MHV (mouse hepatitis virus) infection reduces the incidence of diabetes mellitus in non-obese diabetic mice", *Diabetologia* **34**, 2–5.

Zaccone, P., Fehervari, Z., Jones, F.M., Sidobre, S., Kronenberg, M., Dunne, D.W. and Cooke, A. (2003) "*Schistosoma mansoni* antigens modulate the activity of the innate immune response and prevent onset of type 1 diabetes", *Eur. J. Immunol.* **33**, 1439–1449.

